# Age differences in the influence of self-esteem and emotional regulation on memory

**DOI:** 10.3389/fpsyg.2024.1346693

**Published:** 2024-09-05

**Authors:** Yaffa Doron, David Anaki

**Affiliations:** ^1^Department of Psychology, Bar-Ilan University, Ramat-Gan, Israel; ^2^The Leslie and Susan Gonda (Goldschmied) Multidisciplinary Brain Research Center, Bar-Ilan University, Ramat-Gan, Israel

**Keywords:** state self-esteem, emotion regulation, old-adults, distraction, reappraisal, memory

## Abstract

Emotion regulation (ER) comprises the processes that recognize, monitor, evaluate, and modify emotional reactions. Although ER refers to events that stem from internal or external situations, few studies have examined the effect of intrinsic emotional states on ER processes deployed on external stimuli. The present research investigated, in old- vs. young adults, the influence of state self-esteem (internal condition) in employing ER strategies while implicitly memorizing negative-valence pictures (external condition). State self-esteem was manipulated by giving random feedback (positive/negative) to a demanding cognitive task. Participants then saw 20 photographs and were asked to reappraise half of them and distract themselves from the other half. They were given a recognition task following a short delay with 20 old photographs and 20 new ones. Results indicated that young people with high self-esteem remembered the reappraised pictures better than the distracted ones. In the low self-esteem state, young adults remembered the distracted photographs better than the reappraised ones. In contrast, in old adults low self-esteem resulted in better recognition than high self-esteem, regardless of the regulation strategy. Thus, only among young participants did emotion regulation strategies moderate the effect of self-esteem on memory for negative emotional images. These findings highlight the intricate interplay between different ER strategies and their relative importance across various stages of life.

## Introduction

While emotions are essential in our everyday lives, regulating these emotions is just as crucial. Emotional regulation (ER) is a person’s mechanism for controlling the emotional system. ER allows us to direct, balance, and manage our emotions, to change our perception of emotional experiences and, consequently, our behavior in a particular situation (Gross, 1998). Diverse emotion regulation strategies have been identified (for review, see Naragon-Gainey et al., 2017); however, not all of them are equally effective, and a person should examine what strategy is the most efficient for him in every situation.

### Emotional regulation strategies

Emotional information processing is modified at two major cognitive stages: early and late. At the early stage regulation can be accomplished by disengaging from emotional information processing before it undergoes elaborated processing in working memory. The classic strategy for this stage is distraction, which involves diverting attention away from an emotional situation by loading working memory with independent, neutral contents (van Dillen and Koole, 2007; Thiruchselvam et al., 2011). Emotional regulation at a late stage is achieved by engagement with incoming emotional information before it determines behavior. The classic strategy for this stage is reappraisal, which alters the impact of emotion by either changing the way a situation is considered or by re-evaluating an emotional stimulus (e.g., Sheppes and Levin, 2013). Reappraisal, relative to distraction, is more effective in reducing the emotional experience (McRae et al., 2010). Sheppes and Levin (2013) found that participants prefer reappraisal over distraction under low negative intensity situations, presumably because reappraisal can successfully modulate immediate emotional responses and provide long-term adaptation. However, under high negative intensity situations, participants prefer distraction over reappraisal because only distraction can successfully block emotional information before it gathers force.

Reappraisal is a complex mental activity which requires executive functions to a greater extent than distraction. These abilities include working memory capacities, set-shifting abilities, and inhibition (Miyake et al., 2000). These facets are incorporated under a single factor, which reflects the ability to activate and maintain task-relevant information and goals (Friedman and Miyake, 2017). Both single facets and the general factor are correlated to reappraisal (McRae et al., 2012; Toh and Yang, 2024).

### Self-esteem and emotion regulation

Several factors determine the type of ER that is being used. One of them is self-esteem. Self-esteem is the set of qualities, beliefs, and opinions about our values, abilities, skills, and status in society, and is positively or negatively oriented toward the self (Blascovich and Tomaka, 1991; Heatherton and Wyland, 2003). People with low self-esteem tend to experience negative emotions more often than those with higher self-esteem (Taylor and Brown, 1988). Therefore, it is essential to regulate negative emotions, especially in individuals with low self-esteem. Self-esteem can also be characterized as a trait or state (Heatherton and Polivy, 1991). While trait self-esteem represents a stable evaluation of the self, state self-esteem is a context-specific state of self-worth that can fluctuate in reaction to situational factors (Crocker and Wolfe, 2001; Rosi et al., 2019; Braun et al., 2020). Rosi et al. (2019) showed that manipulation through positive/negative (success/failure) feedback affected state self-esteem, so success has caused an increase in state self-esteem, and failure caused a decrease in it. Trait self-esteem also influences an individual’s choice of emotional regulation strategy. Shafir et al. (2017) found that participants with lower self-esteem reacted defensively to the threat of failure by seeking short-term relief via distraction over the long-term benefit of reappraisal. In contrast, participants with high self-esteem were less affected and showed no clear preference in choosing a strategy for emotional regulation.

### Effects of emotion regulation and self-esteem on memory

Memory is influenced by both ER and self-esteem. For example, when faced with a distressing situation, individuals often attempt to assess and regulate their negative emotions. By doing so, they change their emotional reaction to the adverse event and alter how the event is encoded in memory. That is, regulation strategies affect memory encoding. Hayes et al. (2010) studied the neural mechanisms leading to memory formation during emotional regulation. They found that re-evaluation contributed to decreasing negative emotion and memory empowerment. Specifically, brain imaging showed that successful coding during reappraisal was associated with activity in brain areas related to emotion and memory processing. This study provides evidence for the role of reappraisal in enhancing emotional and memory processes by changing the way stimuli are encoded. Yeh et al. (2020) also examined how cognitive reassessment affects emotional experience. They found that reappraisal improved memory and cognition (relative to passive viewing) and decreased negative emotional experiences. Other studies found good memory for items observed during reappraisal (Dillon et al., 2007; Martins et al., 2014) and weaker memory for images seen during distraction (Sheppes and Meiran, 2007). The conventional explanation for these findings is that reappraisal involves deeper processing of the emotional meaning of the stimulus. In contrast, distraction requires less attention and, therefore, its coding of emotional meaning is shallower (Craik and Lockhart, 1972).

Self-esteem also affects memory by creating a bias in an individual’s memory for his successes and failures (Demiray and Freund, 2017). Consequently, individuals with high trait self-esteem remember experiences more positively, while those with low self-esteem remember them more negatively (Christensen et al., 2003). There are two explanations for these biases in memory. The first is according to the mood-congruence model, which refers to the information retrieval stage. According to this explanation, activating one of the characteristics of self-image - such as the perception of positivity and negativity of the self (self-liking) or the understanding of the person’s efficacy (self-competence) - creates a situation that allows for better retrieval of the experience or the event that matches the person’s current emotions (Faul and LaBar, 2023). Thus, those with high self-esteem will enjoy improved memory for positive information and vice versa in individuals with low self-image. The second explanation is based on the relevance model – how relevant the event is to a person. This explanation refers to the coding phase, according to which activating one of the self-image characteristics mentioned above leads to better coding and memory of negative content among people with low self-image compared to those with high self-image (Tafarodi et al., 2003). The effects of ER and self-esteem on memory were studied separately. No study, to date, has examined their combined effects and whether they contribute separately or jointly to memory performance. Previous research suggests that the relationship may be interactive rather than additive: Shafir et al. (2017) found that participants with low self-esteem adopt a defensive manner and prefer distraction as a regulatory technique. As a result, better memory will be observed after distraction. Moreover, the mood-congruence and relevance models predict better memory in low self-esteem participants. In contrast, those with high self-esteem do not show clear preference. Therefore, reappraisal may be a better conduit for enhanced memory due to its elaborated encoding relative to distraction.

### Effects of age on emotion regulation and self-esteem

Finally, age is critical in ER (Nolen-Hoeksema and Aldao, 2011) and self-esteem (Rosi et al., 2019). Regarding ER, studies found a preference among old adults for distraction over reappraisal, while young adults showed the opposite picture, especially when the negative stimuli were of low intensity (Sheppes et al., 2011; Scheibe et al., 2015, but see Hamilton and Allard, 2021; Martins et al., 2014, 2016). This preference was attributed to age differences in cognitive capacities, which decrease reappraisal efficacy (Winecoff et al., 2011). This account is supported by physiological indices showing more pupil dilation in older but not young adults, indicating more cognitive effort during reappraisal than distraction (Martins et al., 2018). Imaging results also suggest that older adults activate, to a lesser extent than young adults, areas in the prefrontal cortex (PFC), which are related to processes that support emotion regulation. PFC’s lower activation may be related to adults’ limited ability to use a re-evaluation strategy (Allard and Kensinger, 2014). Specifically, studies that examined brain activity in young and old adults during emotional regulation suggest that older adults are less effective than their younger counterparts in using reassessment strategies to reduce the negative impact in real time (Tucker et al., 2012). This assumption follows the understanding that assessment is based on cognitive processes within the PFC, which decrease with age. For example, a reduction in the ventrolateral prefrontal cortex (VLPFC) activity is related to difficulties in delaying adverse reactions (Silvers et al., 2013; Lantrip and Huang, 2017).

Regarding the effects of age on self-esteem some studies have shown a decrease in trait self-esteem in old age (e.g., Orth et al., 2018), yet other studies have found that it remains stable (e.g., Wagner et al., 2013). State self-esteem has also been reported to be more stable and higher with age (Meier et al., 2011). However, others have shown that older and young adults were affected to the same extent by experiences of successes and failures (Rosi et al., 2019). The different results of the two studies may stem from the different approaches used to measure self-esteem: While the former study used a diary study design where participants reported their daily self-esteem for 25 days, the latter was based on a single success-failure manipulation in a demanding cognitive task.

### The present research

The objective of the present research was to investigate how ER (distraction vs. reappraisal), state self-esteem (high vs. low), and age (young adults vs. older adults) affect memory. To the best of our knowledge, the conjoint impact of these variables on memory has not been examined. In the current study, we presented a cognitively demanding task to young and old adults. We gave them random feedback– either positive, to increase their self-esteem, or negative, to reduce it. Then, we presented negative emotional images and instructed the participants to implement one of two regulation strategies, reappraisal or distraction, to deal with the negative emotions of the images (learning phase). Participants were not told that their memory would be later probed. During the test phase, we presented them (randomly) with negative emotional images – half of them from the learning phase and the other half new photos – and asked them to identify which images were old or new.

We examined several hypotheses in the present research: First, we expected to find significant age, feedback, and ER main effects so that young participants, individuals with high state self-esteem, and the use of reappraisal ER strategy would yield better memory performance than older adults, people low in state self-esteem and distraction ER strategy. Second, based on the findings that showed comparable effects of failure-success feedback in young- and old adults, we hypothesized that low-state self-esteem would yield lower memory accuracy than high-state self-esteem for both groups. However, since we could not overlook other findings indicating that with increasing age, state self-esteem is more stable and less sensitive to relevant life events, our hypothesis regarding the age X self-esteem interaction was not definitive. Third, we expected to find a self-esteem X ER interaction so that following positive feedback, a reappraisal strategy will result in better memory for stimuli than a distraction. However, following negative feedback, an ER strategy of distraction will lead to better memory. Finally, we hypothesized that this self-esteem X ER interaction will be modified by age and appear only in young adults. Since self-esteem is less susceptible to manipulations in older adults, and reappraisal requires more cognitive efforts in this population, we expected that distraction would lead to better memory than reappraisal, regardless of feedback.

## Materials and methods

### Participants

The study included a total of 132 participants. Of them 70 were young adults (*M* = 30.94, SD = 7.44) and 62 older adults (over 65 years old, *M* = 77.03, SD = 7.64). G*power software was used to determine the sample size *a priori* (Faul et al., 2009). A mixed-design ANOVA (age group [between-subjects], feedback [between-subjects], and emotion regulation strategy [within-subject]) and a small-medium effect size (*f* = 0.15, α error = 0.05, power = 0.80), required a total sample size of 128. Participants were recruited through random personal contacts, the recruitment database of Bar-Ilan University, senior adult centers, and social networks (e.g., Facebook). Participants were reimbursed with course credit or small financial compensation (~USD $3). None of them reported any brain injury (accident, illness, stroke) in the past. Participants in each age group were randomly allocated to either the failure or the success condition. The study was conducted following the Helsinki Declaration and approved by the Institutional Review Board of the Department of Psychology at Bar-Ilan University.

### Materials

*Questionnaire.* Rosenberg Self-Esteem Scale (RSES; Rosenberg, 1965). The RSES is a 10-item single-dimensional scale that measures positive and negative feelings about the self. Each item was answered on a 5-point Likert-type scale ranging from 1 (strongly agree) to 5 (strongly disagree). The current study used the Hebrew version taken from Vardi’s (2006) study. Cronbach’s alpha in Vardi’s (2006) study was 0.75. In the current study it was 0.86.

*Stimuli.* We used 40 negative emotional images from the International Affective Picture Database System (IAPS, Lang et al., 1997). Mean valence ratings were 2.88 (SD = 0.88) while mean arousal ratings were 6.16 (SD = 0.67). The 40 stimuli consisted of 20 pairs of similar images. The first image in each pair was designated as A (i.e., A1-A20), and the second image in each pair was defined as B (i.e., B1-B20). The lists were divided into sub-lists (i.e., A1-A10, A11-A20, B1-B10, B11-B20). Two sub-lists were presented in the emotional regulation task (i.e., 2nd stage detailed below). Participants applied reappraisal on the first ten images (e.g., A1 to A10 or B1 to B10) and distraction on the second 10 images (e.g., A11 to A20 or B11 to B20). In the recognition memory task (i.e., 3rd stage detailed below) all 4 sub-lists were presented, namely the old ones (A1-A20) and new ones (B1 to B20). Both the lists and the sub-lists were counterbalanced in the 2nd stage. The four sub-lists did not differ in valence ratings, *F* (3,36) = 0.66, *p* > 0.58. Regarding arousal ratings, the analysis was significant, with *F* (3,36) = 3.64, *p* = 0.02. However, post-hoc analysis with Bonferroni correction did not yield any significant difference between the four sub-lists.

**Figure 1:**
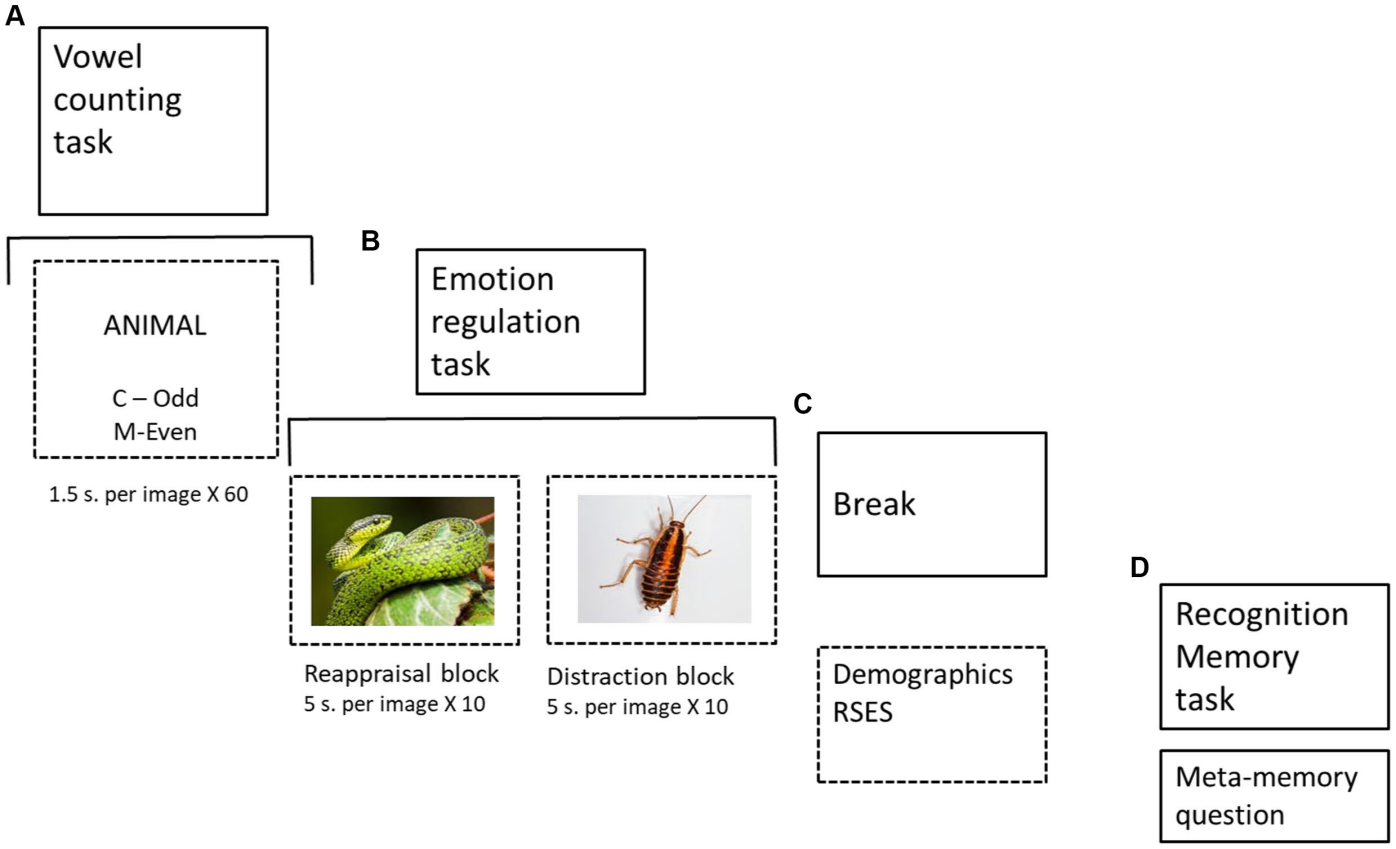
Experiment structure. **(A)** Vowel counting task (followed by random feedback); **(B)** Implementation of two emotion regulation strategies in two blocks (10 images for distraction and reappraisal each); **(C)** Demographic questionnaire and self-report self-esteem questionnaire (i.e., RSES); **(D)** Memory test (40 images, 20 old and 20 new) with a meta-memory question (Photos by Erik Karits and Alfonso Castro on Unsplash).

*Tasks.* Three cognitive tasks were designed. The first task was a Hebrew vowel counting task: Each trial started with a 1,000 ms fixation cross. It was followed by a word presented for 1,500 ms. Within this time window, the participant had to count the number of vowels in the word and press the “N” key if the number of those was odd and the “C” key if the number was even. Sixty words were presented in this task. At the end of the task, participants were randomly given a positive (“Well done, congratulations! You succeeded in responding correctly for most of the words. Most of the subjects did not reach the level you reached”) or negative (“Too bad, you failed on most of the words. Most of the subjects reached a higher level”) to increase or reduce, respectively, their state self-esteem (Figure 1). Note that participants could not independently assess their performance due to the short length of the trial.

The second task was an emotion regulation task. Participants were instructed how to implement two emotional regulation strategies – reappraisal (e.g., “Think that the image is fabricated, or give a more positive meaning to the negative detail of the image”) or distraction [e.g., “Distract yourself from the emotional part in a picture by referring to the non-emotional details in it” (Yan et al., 2018)]. They were shown two pictures as examples of the different emotion regulation strategies. Then, 20 negative pictures from the IAPS database were displayed on the screen for 5 s (following Sheppes et al., 2014 and van Reekum et al., 2007). The images were presented in two blocks—10 images for each strategy. The block order and images were counterbalanced across participants. The images in each block were shown randomly. Participants were asked to say aloud their thoughts while applying the different strategies.

The third task was a recognition memory assignment. Forty pictures (20 old, 20 new) were shown to the participants, who had to indicate whether the picture was presented previously in the emotion regulation task. Memory performance was tested in a two-alternative forced choice (2AFC) recognition task (new/old images). The pictures were shown until the response. The two sets of pictures were similar and featured comparable stimuli. At the end of the task, participants were asked a meta-memory question: which strategy aided them in memorizing the images more?

### Procedure

Upon arrival, participants gave written informed consent. They first performed the vowel counting task, and at the end, they received random feedback, positive or negative. Then, they performed the emotion regulation task. This task was followed by a short break during which participants were asked to fill out a demographic questionnaire and the RSES. Finally, the participants performed the recognition memory task. Only then participants were aware that their memory would be assessed. Participants were debriefed upon completing the study’s objectives.

## Results

To examine the study’s hypotheses, we conducted three-way mixed-designs ANOVAs, with emotional regulation strategy (reappraisal vs. distraction) as a within-subjects variable and feedback (positive vs. negative) and age group (young vs. old adults) as between-subjects variables. Trait self-esteem was added as a covariate to the ANOVAs since older adults had higher self-esteem scores than young adults (*M* = 4.34, *SD* = 0.47 vs. *M* = 3.96, *SD* = 0.72, respectively, *t* (130) = 3.45, *p* < 0.001). Two dependent variables were analyzed in the present study: the first was the hit rate, namely, the number of images identified correctly (i.e., when a participant answered “old” to an old image, Maximum correct answers = 10 for each ER strategy). The second dependent variable was the d-prime (*d’*) sensitivity index calculated as z (hits) − z (false alarms). This index is based on the signal detection theory (SDT; Macmillan and Creelman, 2004) and considers target sensitivity while minimizing false alarms. Follow-up analyses were performed using two-way ANOVAs and *t-tests*.

### Hits

The main effect of emotional regulation strategy was significant, with higher accuracy of reappraised images than distracted ones (*M* = 8.82, *SD* = 2.01, and *M* = 8.78, *SD* = 1.88, respectively, *F* (1,127) = 4.55, *p* < 0.05, 𝜂_p_^2^ = 0.04). Additionally, a significant emotion regulation strategy X feedback interaction, *F* (1,127) = 5.66, *p* < 0.05, 𝜂_p_^2^ = 0.04 was obtained. This interaction was qualified by age as indicated by the significant emotion regulation strategy X feedback X age group interaction, *F* (1,127) = 4.02, *p* < 0.05, 𝜂_p_^2^ = 0.03.

**Figure 2:**
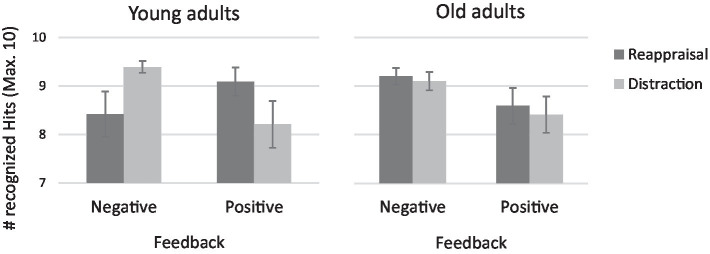
Recognition hits by emotion regulation and feedback for young and old adults.

To examine the source of the three-way interaction, two emotion regulation strategy X feedback ANOVAs were conducted for young and old adults separately (see Figure 2). Among the young adults the emotion regulation strategy X feedback interaction was significant, *F* (1,67) = 6.65, *p* < 0.01, 𝜂_p_^2^ = 0.09. Further analyses showed that when feedback was positive, reappraisal enhanced memory more than distraction, *t* (33) = 1.85, *p* = 0.07, but when feedback was negative, distraction produced better memory than reappraisal, *t* (35) = 2.13, *p* < 0.05, as according to our hypothesis. In contrast, among old adults, negative feedback resulted in better recall, regardless of the type of emotion regulation, *F* (1,59) = 3.11, *p* = 0.08, 𝜂_p_^2^ = 0.05.

### *d’* analysis

The three-way mixed-designs ANOVA yielded a significant main effect of feedback, *F* (1,127) = 3.82, *p* = 0.05, 𝜂_p_^2^ = 0.03 (*d’* positive: *M* = 2.24, *SD* = 0.71, *d’* negative: *M* = 2.48, *SD* = 0.70). Additionally, results revealed a main effect of age, *F* (1,127) = 4.78, *p* < 0.05, 𝜂_p_^2^ = 0.04, so that young adults showed high memory scores than old adults (*d’* young adults: *M* = 2.50, *SD* = 0.71, *d’* old adults: *M* = 2.22, *SD* = 0.72). Similarly, the main effect of emotional regulation strategy was significant, with higher accuracy of reappraised images than distracted ones (*M* = 2.37, *SD* = 0.80, and *M* = 2.34, *SD* = 0.79, respectively, *F* (1,127) = 3.78, *p* = 0.05, 𝜂_p_^2^ = 0.03).

**Figure 3:**
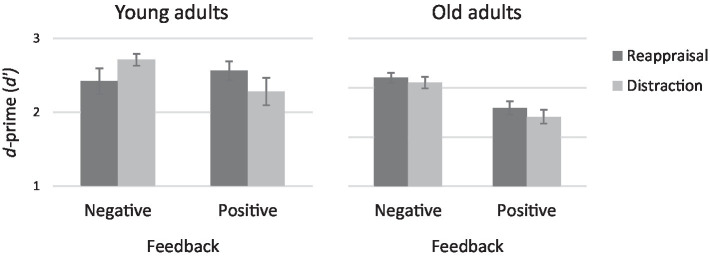
Recognition *d*-prime *(d’)* by emotion regulation and feedback for young and old adults.

As in the Hit analysis the emotion regulation strategy X feedback interaction was significant, *F* (1,127) = 5.05, *p* < 0.05, 𝜂_p_^2^ = 0.04. Moreover, in line with our third hypothesis, this latter interaction was qualified by age, *F* (1,127) = 3.38, *p = 0*.07, 𝜂_p_^2^ = 0.03. To examine the source of the three-way interaction, two separate two-way ANOVA were conducted for young and old adults separately, with the independent variables being emotional regulation strategy and feedback (see Figure 3).

In young adults the two-way interaction was significant, *F* (1,67) = 5.88, *p* < 0.05, 𝜂_p_^2^ = 0.08; Simple main effects showed that when feedback was positive, reappraisal produced better memory than distraction (*t* (32) = 1.78, *p* = 0.08), but when feedback was negative, results were reversed so that distraction produced better memory than reappraisal, *t* (35) = 1.99, *p* < 0.06. In contrast to young adults, old adults showed a significant main effect of feedback, *F* (1,59) = 4.37, *p* < 0.05, 𝜂_p_^2^ = 0.07; Negative feedback brought to better memory than positive feedback, regardless of regulation strategy.

Analysis of the participants’ self-report on their preferred strategy showed that 28/34 (82%) of the young adults who received positive feedback told their preference for reappraisal over distraction. Among the recipients of negative feedback, an equal number of participants reported one of the two strategies (reappraisal: *n* = 17 [47%]; distraction: *n* = 19 [53%]). Among the old adults, there was a preference of 62% (20/32) for distraction among those who received positive feedback and 57% (17/30) among those who received negative feedback. A chi-square test of independence was performed to evaluate the relationship between feedback and preferred strategy. The relationship between these variables was significant in young adults, χ^2^(1) = 9.40, *p* < 0.002, but not in older adults, χ^2^(1) =0.22, *p* > 0.64.

## Discussion

The present study examined the effect of emotion regulation and state self-esteem on the memory of negative emotional images in young and older adults. Our first hypothesis – that significant main effects would be revealed for age, ER, and state self-esteem, was partially confirmed: Young adults remembered (in the d’ prime dependent measure) the pictures better than older adults. The reappraisal strategy enhanced memory compared to distraction (in both dependent measures). Finally, feedback type influenced memory: Better memory (in d’ prime) was exhibited in the negative feedback condition than in the positive one. This latter finding is contrary to our original assumption.

Our second and third hypotheses were also significant. Focusing on our central hypothesis, we found a significant interaction between emotion regulation and state self-esteem, modulated by age: Young adults with high self-esteem (due to positive feedback) remembered better when they used a reappraisal strategy than a distraction strategy. In contrast, young adults who received negative feedback and were, therefore, with low self-esteem recognized distracted images more than reappraised ones. This pattern of results was not seen among old adults, where we found that negative feedback resulted in better memory than positive feedback, regardless of the regulation strategy. Thus, our hypothesis was confirmed for young adults but not for old adults, where we predicted that the ER strategy, but not state self-esteem, would influence memory.

Several studies have shown that reappraisal improves memory compared to distraction (e.g., Richards and Gross, 2000; Dillon et al., 2007). This relative enhancement is particularly evident in situation-focused reappraisal instructions used in the present study (Willroth and Hilimire, 2016). One possible account for this is that reappraisal requires effortful processing and elaboration of the stimulus details, resulting in more robust and stable memory traces. This account is in line with previous research in memory and, in particular, with the levels of processing model (Craik and Lockhart, 1972), which claims that deeper rather than shallow encoding leads to better memory.

However, reappraisal has not yielded better memory in all situations. The present study shows that when participants received negative feedback, their memory performance was better when they used a distraction strategy. This result can be explained by the fact that failure (negative feedback) harms young adults’ self-esteem, so they lose their motivation to implement an emotional regulation that requires cognitive effort (such as reappraisal; Shafir et al., 2015). In such cases, distraction is more effective for them because it minimizes the emotional experience in the short term (Shafir et al., 2017). Being released from negative emotions, they can invest their cognitive resources to focus on the non-emotional aspects of the images they see.

This pattern of behavior did not appear in old adults, where we found that negative feedback evoked better memory, regardless of the strategy pattern of regulation. These findings show that in contrast to our initial hypothesis, the harm to self-esteem (due to the negative feedback) affects old adults’ motivation to prove their ability to cope with cognitive tasks, such as ER. Therefore, they strive to achieve good results, even more than when receiving positive feedback. Studies show that old adults are motivated to invest cognitive effort in what is most important to them in the immediate term (e.g., Carstensen, 2006). Thus, after receiving negative feedback in the present study, they were motivated to perform the regulation task satisfactorily to improve their negative feelings.

Additionally, the finding that low self-esteem in old adults promotes better memory than high self-esteem may be due to adults’ reference to a younger subjective age, namely, feeling younger than their chronological age (Gana et al., 2004; Rubin and Berntsen, 2006; Stephan et al., 2016). Experimental studies have revealed that younger subjective age is a self-defense strategy that manifests itself in response to age-dependent negative stereotypes (Levy, 2009; Weiss and Freund, 2012; Weiss and Lang, 2012; Chen et al., 2018). Thus, people who adopt a younger subjective age may be less sensitive to the harmful effects of negative stereotypes on memory performance (Levy, 2009). Indeed, younger perception of age (relative to actual age) is associated with higher self-efficacy in memory functions (Schafer and Shippee, 2010; Stephan et al., 2011), which contributes to maintaining memory performance in old age (Valentijn et al., 2006). In the present study, the negative feedback given to older adults may have encouraged them to “justify” their younger (subjective) sense of age and invest effort in the emotion regulation task. Succeeding in this task strengthened their belief in coping with other cognitive tasks and consequently improved their memory. Note that this latter account explains why these results were found in old adults only.

While the type of efficient ER strategy in young adults was context-dependent (e.g., type of feedback), no such dependence was found in old adults, where appraisal and distraction were equally efficient. Some studies (e.g., Urry and Gross, 2010) suggested that older people prefer ER strategies that require less cognitive effort, like suppression. In contrast, other studies (e.g., Gerolimatos and Edelstein, 2012) showed a higher preference for reappraisal. A recent systematic review (Allen and Windsor, 2019) could not clarify the mixed results in ER preference and attributed them to different moderator variables, such as individual differences and situational factors.

At the end of the experiment, we asked participants to report which emotion regulation strategy was more beneficial to their memory. Young adults who received positive feedback stated that reappraisal was more advantageous to memory than distraction – a result consistent with their actual memory performance, as was found in previous studies (Hayes et al., 2010). However, when the feedback was negative, there was no significant preference for one of the strategies (although actual results showed that they remembered better when applying the distraction strategy). Among old adults, it was found that in any condition of the state self-esteem (positive and negative feedback), participants preferred distraction. Still, their memory findings showed no difference between the two regulation patterns. More research is required to examine the role of meta-cognitive processes in general and meta-memory processes in particular in assessing their interactive role with ER procedures.

### Limitations and future directions

A primary limitation in the present study is whether the observed memory effects resulted from state self-esteem or other alternative explanations. Although previous studies examined the impact of success-failure manipulations on self-esteem as we did in our research (e.g., Guay et al., 2008), this manipulation could have impacted other processes such as self-efficacy or affect. Self-esteem, self-efficacy, and affect are strongly correlated (e.g., Joshanloo, 2022) and sometimes used interchangeably (e.g., Nummenmaa and Niemi, 2004). In addition, self-esteem is defined by both affective and efficacy aspects. Thus, due to their considerable overlap, it may be hard to tease apart these concepts and examine their contribution to memory recognition directly or with age and ER as moderators.

Several research venues are recommended for follow-up studies. First, memory measurement was immediate, so it was relatively easy to remember which images had already appeared at the learning stage. It is advisable to examine extended long-term memory in future research. Also, in the present study, we chose negative high-arousal images. Future studies should determine whether the arousal level is an additional factor influencing ER X self-esteem interaction on memory. Finally, a finer distinction between old- and very old adults is recommended. Our findings suggest that negative feedback significantly improved memory performance in older adults. However, chronological age may enforce boundaries on this capability, and feedback may induce varying motivations at different ages.

## Conclusion

The current study shows that state self-esteem and ER have a significant yet differential role in predicting young- and old-adult memory. In young adults, it influences the ER strategy used, leading to memory optimization when (a) reappraisal is coupled with high self-esteem and (b) when distraction is coupled with low self-esteem. In old adults, self-esteem is the sole predictor of enhanced memory. Thus, self-image is essential for adults, as it affects their motivation to prove their ability to cope with cognitive tasks at their age. These results accentuate the different dynamics that characterize ER processes in young and old age.

## Data availability statement

The raw data supporting the conclusions of this article will be made available by the authors, without undue reservation.

## Ethics statement

The study was approved by the ethics committee of the Psychology department at Bar-Ilan University. The studies were conducted in accordance with the local legislation and institutional requirements. The participants provided their written informed consent to participate in this study.

## Author contributions

YD: Conceptualization, Data curation, Formal analysis, Investigation, Methodology, Project administration, Validation, Visualization, Writing – original draft. DA: Conceptualization, Formal analysis, Funding acquisition, Investigation, Methodology, Resources, Software, Supervision, Visualization, Writing – review & editing.
